# A Fast Color Image Encryption Algorithm Using 4-Pixel Feistel Structure

**DOI:** 10.1371/journal.pone.0165937

**Published:** 2016-11-08

**Authors:** Wang Yao, Faguo Wu, Xiao Zhang, Zhiming Zheng, Zhao Wang, Wenhua Wang, Wangjie Qiu

**Affiliations:** 1 Key Laboratory of Mathematics, Informatics and Behavioral Semantics, Ministry of Education, Beijing 100191, China; 2 School of Mathematics and Systems Science, Beihang University, Beijing 100191, China; 3 Sino-French Engineer School (École Centrale de Pékin), Beihang University, Beijing 100191, China; 4 AVIC Economics & Technology Research Establishment, Beijing 100029, China; 5 Educational Equipment Research & Development Center, Ministry of Education, Beijing 100080, China; Lanzhou University of Technology, CHINA

## Abstract

Algorithms using 4-pixel Feistel structure and chaotic systems have been shown to resolve security problems caused by large data capacity and high correlation among pixels for color image encryption. In this paper, a fast color image encryption algorithm based on the modified 4-pixel Feistel structure and multiple chaotic maps is proposed to improve the efficiency of this type of algorithm. Two methods are used. First, a simple round function based on a piecewise linear function and tent map are used to reduce computational cost during each iteration. Second, the 4-pixel Feistel structure reduces round number by changing twist direction securely to help the algorithm proceed efficiently. While a large number of simulation experiments prove its security performance, additional special analysis and a corresponding speed simulation show that these two methods increase the speed of the proposed algorithm (0.15s for a 256*256 color image) to twice that of an algorithm with a similar structure (0.37s for the same size image). Additionally, the method is also faster than other recently proposed algorithms.

## Introduction

With the rapid development of multimedia communications, the efficiency and security of image encryption transmission has become increasingly important. Due to the excellent security properties of chaos, such as ergodicity and sensitivity to initial conditions and parameters, chaos-based image encryption algorithms have attracted more and more attention since they were first proposed by the British mathematician Matthews R. in 1984 [1]. Afterwards, many chaotic image encryption algorithms have been designed based on different chaos maps and structures [2–20]. In particular, due to larger data capacities and higher correlation among pixels, the encryption of color images demand better statistical and diffusion properties in image algorithms than gray images. Thus, color image encryption has recently attracted substantial attention.

Efficiency is a very important factor in the design of chaotic image encryption algorithms. There are some well-known algorithms as examples. All these algorithms were considered safe at the time and gave special attention to their efficiency, yielding successful results. In 2004, Chen et al. proposed a symmetric image encryption scheme that employed the 3D cat map to shuffle the positions of image pixels and used another chaotic map to confuse the relationship between the cipher-image and the plain-image. This algorithm could be used to encrypt a 256*256 image in less than 0.4s [21]. In 2006, Pareek et al. proposed an algorithm that used an external 80-bit secret key and two chaotic logistic maps that could encrypt a 256*256 image in 0.33∼0.39s [22]. In 2013, Fu et al. proposed a very efficient medical image protection scheme based on chaotic maps using a substitution mechanism in the permutation process through a bit-level shuffling algorithm. This algorithm took only 9.5ms to encrypt a 512*512 gray image [23]. However, all three algorithms were broken later. Both Chen’s and Fu’s algorithms were vulnerable to a chosen-plain-text attack [24, 25]. Pareek’s algorithm used logistic maps that have been proven unsafe now. In recent times it has become challenging to find the correct balance of security and efficiency in image encryption algorithms.

Many new thoughts and methods have been introduced to the design of color image encryption algorithms in recent years, as recently as 2015. Liu et al. proposed a new chaotic color image encryption algorithm in which the hash value of the plain image is applied to produce two initial values of the Henon map that generate two pseudo-random sequences [26]. A novel color image encryption with heterogeneous bit-permutation and correlated chaos was proposed by Wang et al. [27]. Murillo-Escobar et al. presented a colour image encryption algorithm based on total plain image characteristics to resist a chosen/known plain image attack, and used a 1D logistic map with optimized distribution to create a fast encryption process [28]. Lang proposed a novel color image encryption method using Color Blend and Chaos Permutation operations in the reality-preserving multiple-parameter fractional Fourier transform domain [29]. Som et al. proposed an algorithm in which the original image is first scrambled using the generalized Arnold cat map to achieve confusion and the scrambled image is then encrypted using chaotic sequences generated by multiple one-dimensional chaotic maps [30]. A perturbed high-dimensional chaos system was designed for image encryption according to Devaney and topological conjugate definition by Tong et al. [31]. The proposed algorithm by Oztruk et al. utilized a Lu-like chaotic system capable of exhibiting both Lorenz-like and Chen-like chaotic system behaviors for different parameter values [32]. We propose an algorithm using 4-pixel Feistel structure and chaotic maps; this algorithm realizes both the security and efficiency needs for a color image [33]. Meanwhile, studies on onset of chaos in discrete nonlinear dynamical systems show potential ways to make selection of chaotic systems and security analysis [34]. Currently, all these algorithms have been shown to be secure, but few are optimized for efficiency.

Feistel structure is a well-known structure for traditional block cipher. Feistel structure don’t need to find inverse functions of encryption round functions for decryptions. In encryption and decryption process, algorithms use the same round functions but Feistel structure in different directions. In this point, it demonstrates that Feistel structure is naturally suitable for chaotic image encryption algorithm design as inverse functions are always difficult to be found for chaotic maps. In this paper, a fast color image encryption algorithm is proposed. This algorithm uses a modified 4-pixel Feistel structure and reduces the round number by changing twist direction in a secure way. It also is shown to improve speed while holding a high security level by utilizing simple round functions based on a piecewise linear function, a tent map is proposed.

## Proposed algorithm

The proposed algorithm can be divided into 3 levels. The basic level utilizes the functions based on multiple chaotic maps. The intermediate level uses the 4-pixel Feistel structure. The top level is the dependent encryption process.

### Functions based on multiple chaotic maps

On the basic level, five chaotic maps or functions are used. Two 3D chaotic systems (Lorenz system as Eq (1) and Chen’s system as Eq (2)) are utilized as key generators to offer round keys for encryption and decryption. Compared with low-dimension maps, the high-dimension chaotic system is more complex with more variables and parameters, which makes algorithm’s key space larger and system variables’ time sequence more erratic and unpredictable. When *p* = 10, *r* = 28 and *t* = 8/3, Lorenz system involves chaotic state and when *a* = 35, *b* = 3 and *c* = 28, Chen’s system is chaotic.

$$\begin{array}{c} \left\{ \begin{array}{c} {\dot{x}}_{1}=p\left(x_{2}-x_{1}\right)\\ {\dot{x}}_{2}=-x_{1}x_{3}+rx_{1}-x_{2}\\ {\dot{x}}_{3}=x_{1}x_{2}-tx_{3} \end{array}\right. \end{array}$$ (1)

where *p* = 10, *r* = 28 and *t* = 8/3.

$$\begin{array}{c} \left\{ \begin{array}{c} {\dot{x}}_{1}=a\left(x_{2}-x_{1}\right)\\ {\dot{x}}_{2}=\left(c-a\right)x_{1}-x_{1}x_{3}+cx_{2}\\ {\dot{x}}_{3}=x_{1}x_{2}-bx_{3} \end{array}\right. \end{array}$$ (2)

where *a* = 35, *b* = 3 and *c* = 28.

With $\left\{x_{1}^{*},x_{2}^{*},x_{3}^{*}\right\}$ and $\left\{x_{4}^{*},x_{5}^{*},x_{6}^{*}\right\}$ as the initial conditions, the Lorenz system and Chen’s system are solved using the 4^*th*^ order Runge-Kutta method with a step size of *h* = 0.001. Solutions {*x*_1_, *x*_2_, *x*_3_} of each system at step *i* are noted as {*x*_1_(*i*), *x*_2_(*i*), *x*_3_(*i*)}.After being initialized *M*_0_ and *N*_0_ times, these two systems generate 6 round keys using Eq (3). Thus, the variables $\left\{x_{1}^{*},x_{2}^{*},x_{3}^{*},x_{4}^{*},x_{5}^{*},x_{6}^{*},M_{0},N_{0}\right\}$ are needed to construct seed keys of the algorithm.

$$\begin{array}{c} \left\{ \begin{array}{c} k_{1}\left(i\right)=\left\lfloor \left(x_{1}\left(i\right)-\left\lfloor x_{1}\left(i\right)\right\rfloor \right)*10^{14}\right\rfloor \;\mathrm{mod}\;256\\ k_{2}\left(i\right)=\left\lfloor \left(x_{2}\left(i\right)-\left\lfloor x_{2}\left(i\right)\right\rfloor \right)*10^{14}\right\rfloor \;\mathrm{mod}\;256\\ k_{3}\left(i\right)=\left\lfloor \left(x_{3}\left(i\right)-\left\lfloor x_{3}\left(i\right)\right\rfloor \right)*10^{14}\right\rfloor \;\mathrm{mod}\;256 \end{array}\right. \end{array}$$ (3)

**Fig 1:**
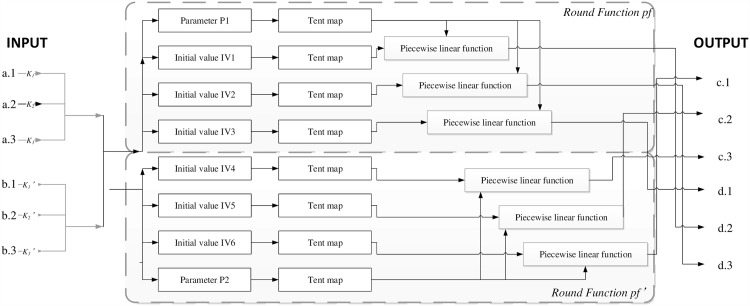
Round function for six colors with two pixels.

Six piecewise linear functions (shown in Eq (4)) and eight tent maps (shown in Eq (5)) are used to construct the round function, shown in Fig 1.

Piecewise linear functions have many good properties in design of chaotic encryption algorithms, such as simplicity in representation, efficiency in implementation, and good dynamical behavior. It has been known that piecewise linear functions are ergodic and have uniform invariant density function on their definition intervals.

The simplicity of the round function contributes to the excellent speed performance of our algorithm.

$$\begin{array}{c} x\left(t+1\right)=\left\{ \begin{array}{cc} x(t)/p & 0\leq x(t)<p\\ \left(x(t)-p)/(0.5-p\right) & p\leq x(t)<0.5\\ \left(1-x(t)-p)/(0.5-p\right) & 0.5\leq x(t)<1-p\\ (1-x(t))/p & 1-p\leq x(t)\leq 1 \end{array}\right. \end{array}$$ (4)

where 0 ≤ *x* ≤ 1 and 0 < *p* < 0.5.

$$\begin{array}{c} x\left(t+1\right)=\left\{ \begin{array}{cc} 2x(t) & 0\leq x(t)<0.5\\ 2(1-x(t)) & 0.5\leq x(t)<1 \end{array}\right. \end{array}$$ (5)

In the round function, the parameters *p*1 and *p*2 are generated with Eqs (6) and (7). Six initial values *IV*1, *IV*2, *IV*3, *IV*4, *IV*5, and *IV*6 are generated from Eq (8).

$$\begin{array}{c} \left\{ \begin{array}{c} p10=(b.1+a.2+b.3)/757+(a.1+b.2+a.3)/761\\ p1=(p10-\lfloor p10\rfloor )/2 \end{array}\right. \end{array}$$ (6)

$$\begin{array}{c} \left\{ \begin{array}{c} p20=(b.1+a.2+a.3)/757+(a.1+b.2+b.3)/761\\ p2=(p20-\lfloor p20\rfloor )/2 \end{array}\right. \end{array}$$ (7)

where *a*.1, *a*.2, *a*.3, *b*.1, *b*.2 and *b*.3 represent six colors that have been handled by the round keys with pixel *a* and pixel *b* (different that those in Fig 1).

$$\begin{array}{c} \left\{ \begin{array}{c} IV1=(a.1+b.3)/(255*2)\\ IV2=(a.2+b.1)/(255*2)\\ IV3=(a.3+b.2)/(255*2)\\ IV4=(a.3+b.1)/(255*2)\\ IV5=(a.1+b.2)/(255*2)\\ IV6=(a.2+b.3)/(255*2) \end{array}\right. \end{array}$$ (8)

where *a*.1, *a*.2, *a*.3, *b*.1, *b*.2 and *b*.3 have the same meanings as those in Eqs (6) and (7).

After parameters *p*1, *p*2 and 6 initial values *IV*1, *IV*2, *IV*3, *IV*4, *IV*5 and *IV*6 have been generated, there are 8 tent maps (as shown in Eq (5)) that are used to improve the chaotic properties. The 6 outputs can then be obtained using six piecewise linear functions, which will consist of 6 cipher colors used to encrypt 2 pixels after the use of Eq (9).

$$\begin{array}{c} a_{e}=\left\lfloor \left(a-\left\lfloor a\right\rfloor \right)*10^{14}\right\rfloor \;\mathrm{mod}\;256 \end{array}$$ (9)

### Modified 4-pixel Feistel structure

At the intermediate level, the modified 4-pixel Feistel structure is used to realize efficient diffusion among 4 pixels at the same time. However, unlike [33], the rounded number of the modified structure reduces while changing its twist direction to remain secure.

**Fig 2:**
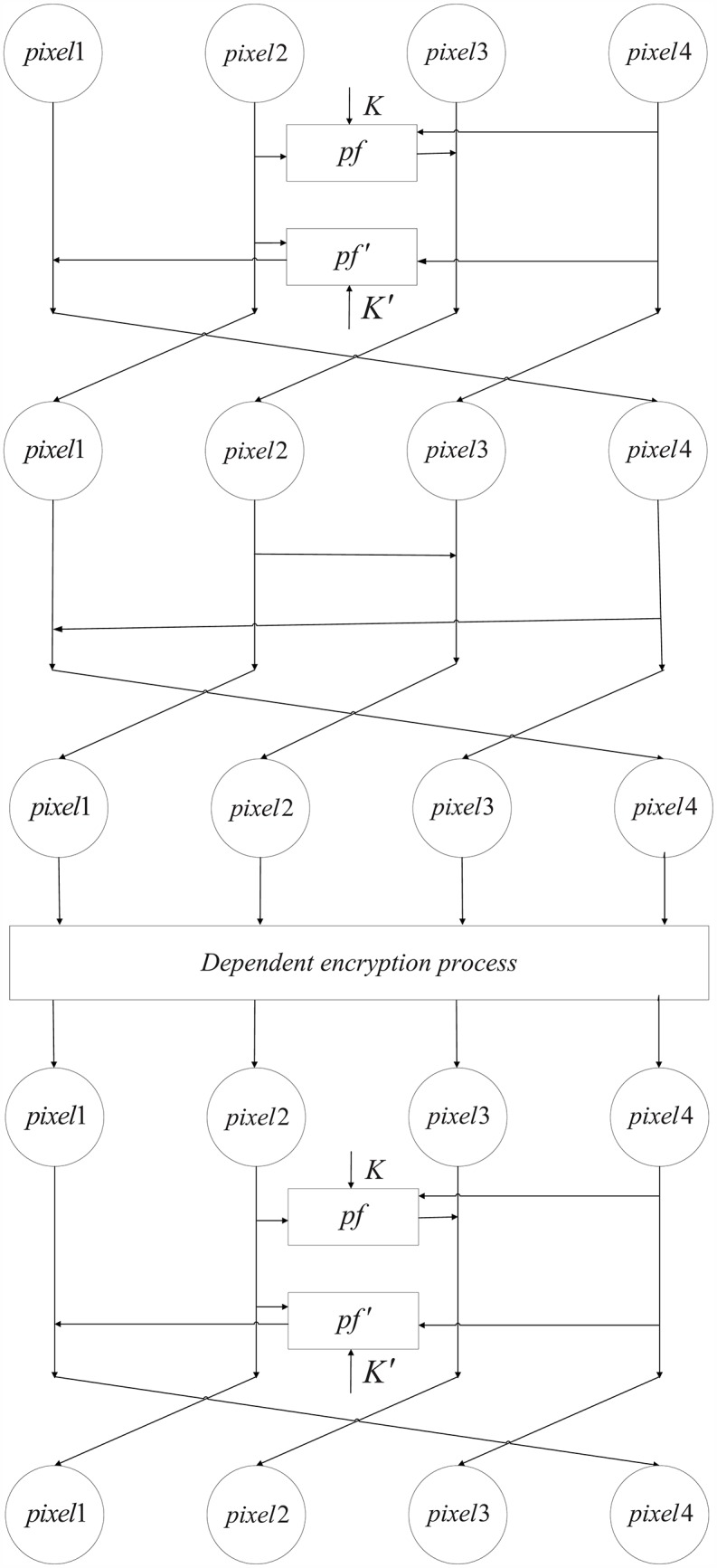
Modified 4-pixel Feistel structure.

The 4-pixel Feistel structure is used twice during the encryption process. However, due to the different functions, each iteration has a different structure, as shown in Fig 2.

When the 4-pixel Feistel structure is first used it has two different rounds. The first round uses a simple round function constructed in the previous subsection, using two group of round keys, *K* = {*k*_1_, *k*_2_, *k*_3_} generated by the Lorenz system and *K*′ = {*k*_4_, *k*_5_, *k*_6_}, generated by Chen’s system. The second round creates diffusion among 4 pixels using x-or operations instead of completing the confusion process used in the first round, as is done in the traditional Feistel method. The confusion in the first iteration is completed by the second iteration of the 1-round 4-pixel Feistel structure.

### Dependent encryption process

At the top level, two instances of dependent encryption processes in different directions are used to extend the effect of changes to all pixels in the image and to resist known-/chosen- plain-text attacks and chosen cipher attacks.

**Fig 3:**
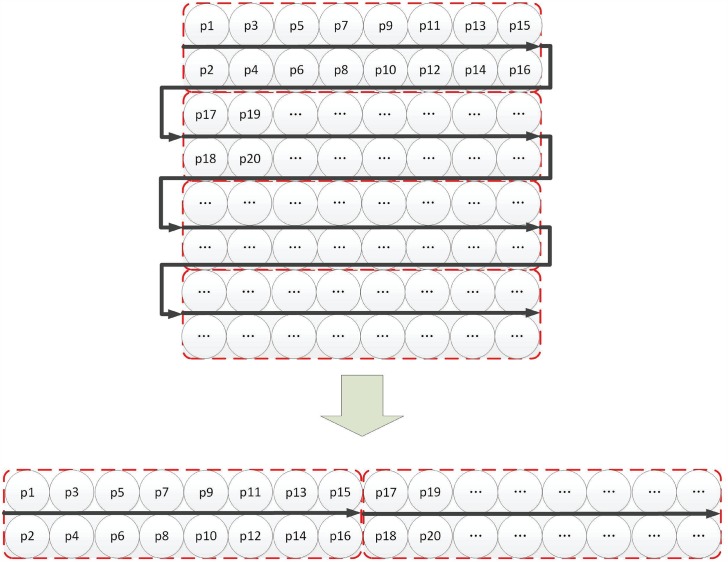
Form changing of dependent encryption.

**Fig 4:**
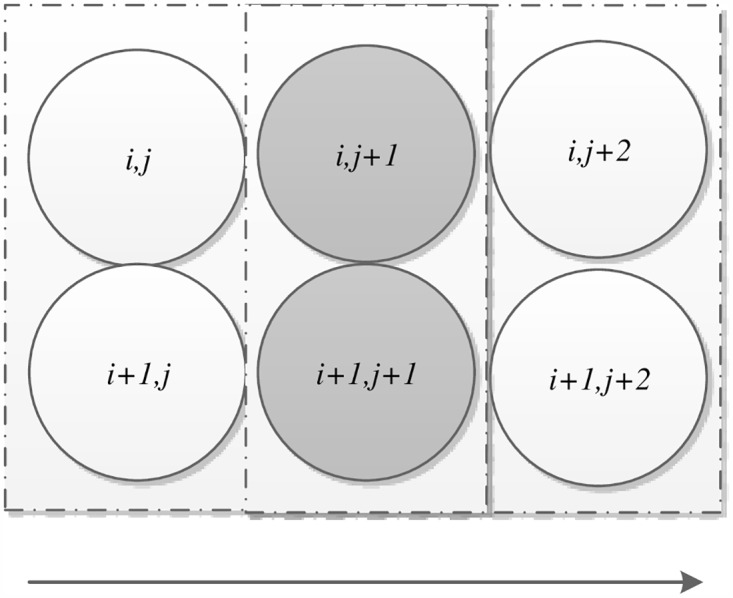
The intersected block.

All the blocks (represented by (*i*, *j*)) are defined by Eq (10), which naturally change the form of the image (shown as Fig 3) and handle the intersected block (shown as Fig 4).

$$\begin{array}{c} \left(\begin{array}{cc} p(i,j) & p(i+2*\lfloor (j+1)/width\rfloor ,(j+1)\;\mathrm{mod}\;width)\\ p(i+1\;\mathrm{mod}\;height,j) & p(i+1+2*\lfloor (j+1)/width\rfloor \;\mathrm{mod}\;height,(j+1)\;\mathrm{mod}\;width) \end{array}\right) \end{array}$$ (10)

where *height* and *width* represent the height and width of the origin image. Here, *i* = 1, 3, 5, …, 1 ≤ *i* ≤ *height*, *j* = 1, 2, …, and 1 ≤ *j* ≤ *width*.

**Fig 5:**
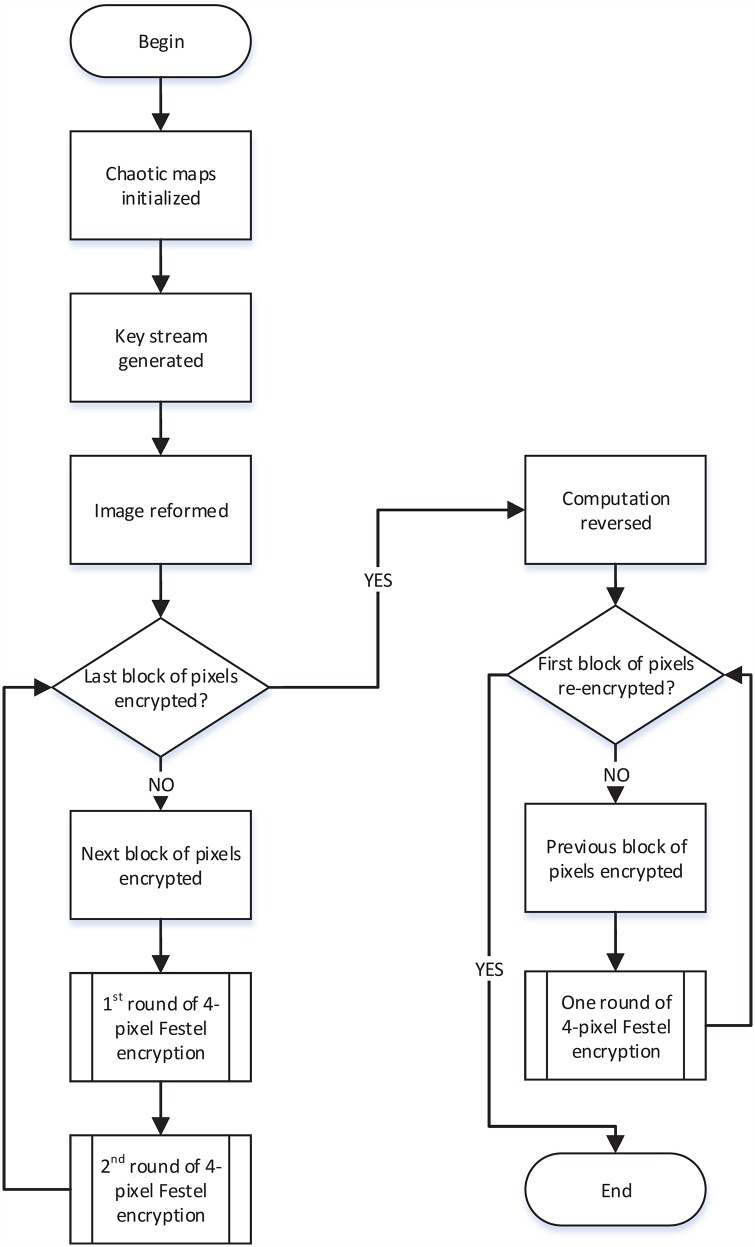
The proposed algorithm.

The proposed algorithm is described by pseudo-code in Alg. 1 and the process and is described by a flow chart in Fig 5.

**Algorithm 1** Proposed algorithm

*Lorenz* *system* *initializes*, *K* *is* *generated*

*Chen*′ *s* *system* *initializes*, *K*′ *is* *generated*

**for** 1 ≤ *i* ≤ *height* − 1 **do**

 **for** 1 ≤ *j* ≤ *width* **do**

  4 − *pixel* *Feistel* *of* *first* *time* *for* *block*(*i*, *j*)

  *j* = *j*+1

 **end for**

 *i* = *i*+2

**end for**

**for** *height* − 1 ≥ *i* ≥ 1 **do**

 **for** *width* ≥ *j* ≥ 1 **do**

  4 − *pixel* *Feistel* *of* *second* *time* *for* *block*(*i*, *j*)

  *j* = *j* − 1

 **end for**

 *i* = *i* − 2

**end for**

## Experimental results and cryptanalysis

In our experiments the following equipment was used, CPU: Intel Core2 Quad CPU Q9500 2.83GHz; Memory: 4.00 GB; Operating system: Windows 7 pro; Coding tool: Visual studio 2012. The experiments include randomness test, histogram analysis, correlation of two adjacent pixels, NPCR and UACI, sensitivity to cipher image, information entropy, key sensitivity, and key space analysis.

**Fig 6:**
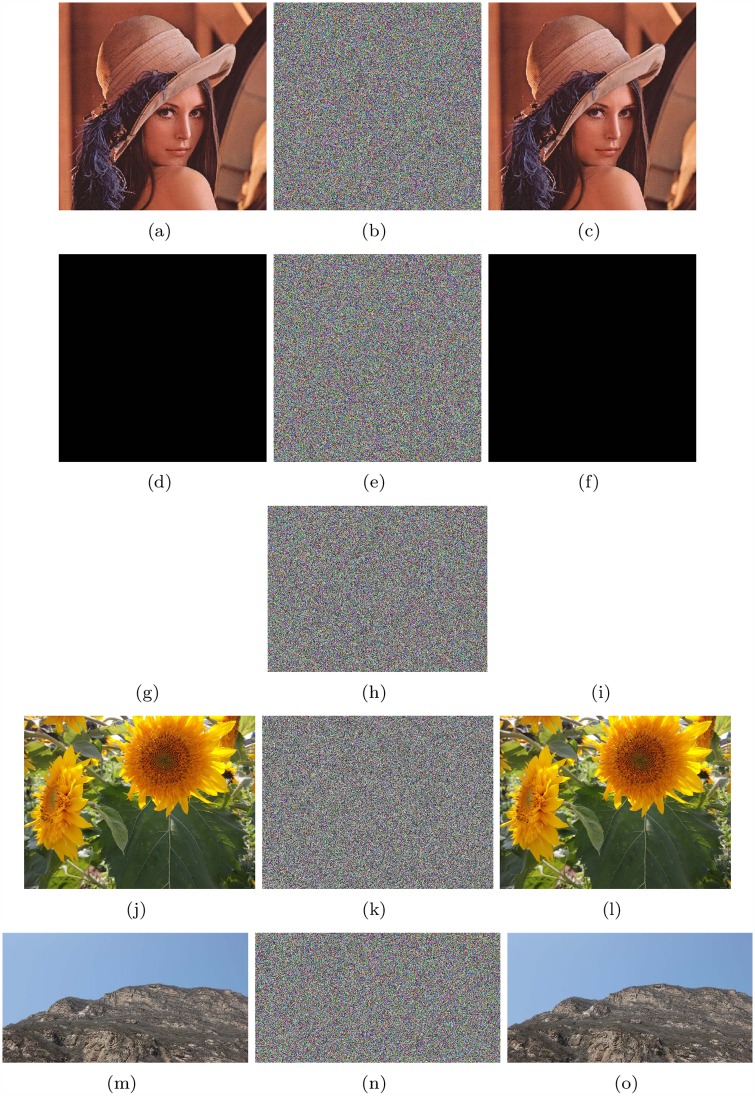
Results of Lena, all-zero image, white, flower and mountain. (a) Plain image Lena; (b) Encrypted image of Lena; (c) Recovered image of Lena; (d) all-zero image; (e)Encrypted image of all-zero image; (f)Recovered image of all-zero image; (g) Plain image white; (h) Encrypted image of white; (i) Recovered image of white; (j) Plain image flower; (k) Encrypted image of flower; (l) Recovered image of flower; (m) Plain image mountain; (n) Encrypted image of mountain; (o) Recovered image of mountain.

The encryption results and corresponding decryption results of Lena (512*512), all-zero image (512*512), white(650*492), flower(1024*768) and mountain(680*360) are shown in Fig 6. Keys $x_{1}^{*}=3.05152212424679$, $x_{2}^{*}=1.58254212245123$, $x_{3}^{*}=15.6238853231785$, $x_{4}^{*}=4.78999921123234$, $x_{5}^{*}=1.98243221252248$, $x_{6}^{*}=14.2532112455785$ are used; *M*_0_ = 20 and *N*_0_ = 30. $x_{1}^{*}$, $x_{2}^{*}$ and $x_{3}^{*}$ are initial values of Lorenz system, while $x_{4}^{*}$, $x_{5}^{*}$ and $x_{6}^{*}$ are initial values of Chen’s system. *M*_0_ and *N*_0_ are the initialization times for these two systems. If it is not otherwise noted, all encryption and decryption examples in our research used the above keys.

### Randomness test

We use sts-2.1.2 test suite offered by NIST(National Institute of Standards and Technology) to test the randomness of our cipher [35]. For each test of sts-2.1.2, there is a predefined threshold for p-value, namely 0.01. When p-value is larger than 0.01, we can conclude the statistical test is passed successfully and the tested sequence is considered as random with 99% confidence. According to results shown in Table 1, we can conclude that our cipher sequence has sufficient randomness.

**Table 1:** Randomness test results.

Test name	p-value	Result
ApproximateEntropy	0.415099	Success
BlockFrequency	0.181519	Success
CumulativeSums-1	0.884862	Success
CumulativeSums-2	0.910365	Success
FFT	0.945089	Success
Frequency	0.972366	Success
LinearComplexity	0.947169	Success
LongestRun	0.523713	Success
NonOverlappingTemplate	0.749729	Success
OverlappingTemplate	0.462382	Success
RandomExcursions	0.290064	Success
RandomExcursionsVariant	0.241670	Success
Rank	0.794957	Success
Runs	0.780795	Success
Serial-1	0.075784	Success
Serial-2	0.032029	Success

### Histogram analysis

**Fig 7:**
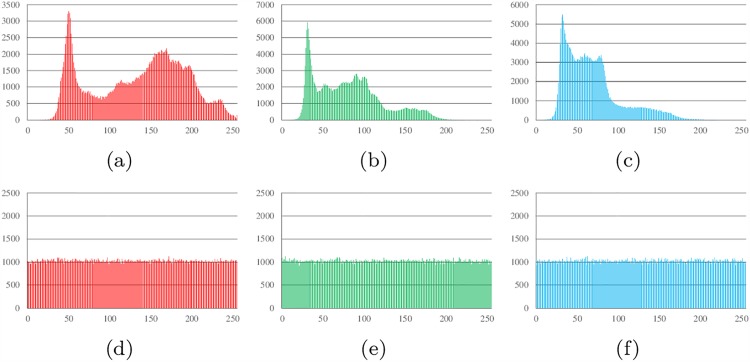
Histogram analysis results of Lena. (a) Histogram of red channel of Lena; (b) Histogram of green channel of Lena; (c) Histogram of blue channel of Lena; (d) Histogram of red channel of cipher; (e)Histogram of green channel of cipher (f)Histogram of blue channel of cipher.

**Fig 8:**
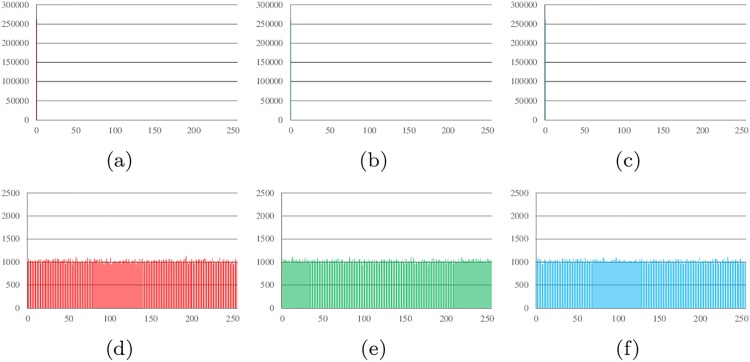
Histogram analysis results of all-zero image. (a) Histogram of red channel of all-zero; (b) Histogram of green channel of all-zero; (c) Histogram of blue channel of all-zero; (d) Histogram of red channel of cipher; (e)Histogram of green channel of cipher (f)Histogram of blue channel of cipher.

**Fig 9:**
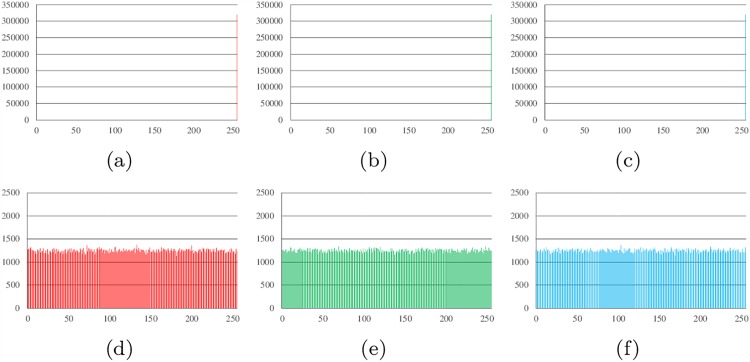
Histogram analysis results of white. (a) Histogram of red channel of white; (b) Histogram of green channel of white; (c) Histogram of blue channel of white; (d) Histogram of red channel of cipher; (e)Histogram of green channel of cipher (f)Histogram of blue channel of cipher.

**Fig 10:**
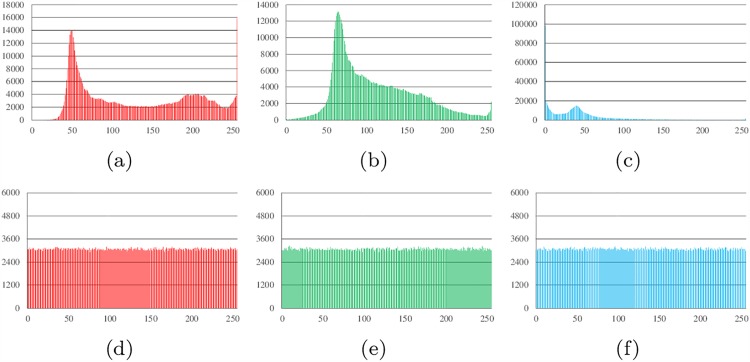
Histogram analysis results of flower. (a) Histogram of red channel of flower; (b) Histogram of green channel of flower; (c) Histogram of blue channel of flower; (d) Histogram of red channel of cipher; (e)Histogram of green channel of cipher (f)Histogram of blue channel of cipher.

**Fig 11:**
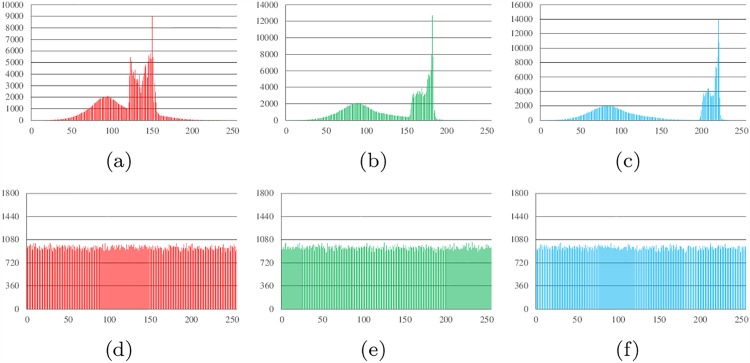
Histogram analysis results of mountain. (a) Histogram of red channel of mountain; (b) Histogram of green channel of mountain; (c) Histogram of blue channel of mountain; (d) Histogram of red channel of cipher; (e)Histogram of green channel of cipher (f)Histogram of blue channel of cipher.

Histograms show the distribution of pixel values in an image. The ideal histogram of a cipher image is uniform. The RGB histogram values of the plain image and the cipher image of Lena, all-zero, white, flower and mountain are shown in Figs 7–11.

It can be found that the histograms of the cipher image are close to uniform. Thus, a frequency analysis cannot be used to break the algorithm.

### Correlation of two adjacent pixels

The correlation of two adjacent pixels can be calculated using the following formula:

$$\begin{array}{c} r_{xy}=\frac{cov(x,y)}{\sqrt{D(x)}\sqrt{D(y)}} \end{array}$$ (11)

where $cov\left(x,y\right)=\frac{1}{N}\sum_{i=1}^{N}\left(x_{i}-E\left(x\right)\right)\left(y_{i}-E\left(y\right)\right)$, $E\left(x\right)=\frac{1}{N}\sum_{i=1}^{N}x_{i}$, $D\left(x\right)=\frac{1}{N}\sum_{i=1}^{N}{\left(x_{i}-E\left(x\right)\right)}^{2}$, and *N* = *height* ∗ *width*.

First, we use this formula to test the correlation between various colors in Lena and its cipher image; this comparison shows that the correlation property between the plain image and the cipher image is good. Results are shown in Table 2.

**Table 2:** Correlation between plain image Lena and its cipher image.

	Red	Green	Blue
*Red*	−0.0028	−0.0001	0.0003
*Green*	−0.0029	0.0010	0.0009
*Blue*	−0.0029	0.0018	0.0014

Then, the correlation between different colors of two adjacent pixels of Lena’s, all-zero’s, white’s, flower’s and mountain’s cipher is tested. Lena’s results are shown in Tables 3–5. The results from the all-zero image are shown in Tables 6–8. All results have been compared with algorithms also using 4-pixel Feistel structure [33]. And the test results of white’s, flower’s and mountain’s cipher are shown in Table 9.

**Table 3:** Horizontal correlation of two adjacent pixel of Lena’s cipher.

		Red	Green	Blue
proposed algorithm	*Red*	0.0006	0.0009	−0.0028
	*Green*	−0.0034	0.0013	0.0001
	*Blue*	−0.0019	−0.0018	−0.0011
algorithm in [33]	*Red*	−0.0010	−0.0016	−0.0013
	*Green*	−0.0016	0.0004	−0.0042
	*Blue*	−0.0036	0.0005	0.0016

**Table 4:** Vertical correlation of two adjacent pixel of Lena’s cipher.

		Red	Green	Blue
proposed algorithm	*Red*	−0.0001	0.0006	0.0023
	*Green*	0.0018	0.0007	−0.0016
	*Blue*	−0.0020	−0.0003	−0.0008
algorithm in [33]	*Red*	0.0010	0.0027	0.0001
	*Green*	−0.0017	0.0038	−0.0006
	*Blue*	0.0011	0.0049	0.0015

**Table 5:** Diagonal correlation of two adjacent pixel of Lena’s cipher.

		Red	Green	Blue
proposed algorithm	*Red*	−0.0004	0.0018	0.0004
	*Green*	−0.0014	−0.0009	−0.0013
	*Blue*	−0.0001	0.0007	−0.0001
algorithm in [33]	*Red*	−0.0001	0.0012	−0.0005
	*Green*	0.0004	−0.0008	0.0031
	*Blue*	−0.0039	0.0031	0.0019

**Table 6:** Horizontal correlation of two adjacent pixel of all-zero’s cipher.

		Red	Green	Blue
proposed algorithm	*Red*	−0.0020	−0.0008	0.0023
	*Green*	−0.0027	0.0026	0.0019
	*Blue*	0.0002	−0.0032	0.0015
algorithm in [33]	*Red*	−0.0014	0.0004	0.0016
	*Green*	0.0011	−0.0035	−0.0018
	*Blue*	0.0002	0.0043	0.0033

**Table 7:** Vertical correlation of two adjacent pixel of all-zero’s cipher.

		Red	Green	Blue
proposed algorithm	*Red*	−0.0004	−0.0005	−0.0011
	*Green*	−0.0003	0.0001	−0.0043
	*Blue*	0.0016	−0.0011	0.0009
algorithm in [33]	*Red*	−0.0012	−0.0025	−0.0012
	*Green*	−0.0033	−0.0004	0.0025
	*Blue*	0.0007	0.0012	−0.0042

**Table 8:** Diagonal correlation of two adjacent pixel of all-zero’s cipher.

		Red	Green	Blue
proposed algorithm	*Red*	0.0051	0.0024	−0.0026
	*Green*	−0.0054	−0.0018	−0.0005
	*Blue*	0.0013	−0.0010	0.0015
algorithm in [33]	*Red*	0.0003	−0.0006	0.0041
	*Green*	0.0011	0.0018	0.0008
	*Blue*	0.0003	0.0016	0.0050

**Table 9:** Correlation of two adjacent pixel of white’s, flower’s and mountain’s cipher.

			Red	Green	Blue
white	Horizontal	*Red*	−0.000606	0.000700	−0.002371
		*Green*	−0.001305	0.002099	−0.001127
		*Blue*	−0.001729	0.003320	0.000485
	Vertical	*Red*	−0.000292	0.000844	−0.000420
		*Green*	0.001426	0.001139	−0.000827
		*Blue*	−0.003630	−0.000348	−0.002165
	Diagonal	*Red*	0.001916	−0.002552	0.001659
		*Green*	−0.001534	0.004926	−0.001456
		*Blue*	0.001086	0.001619	−0.003627
flower	Horizontal	*Red*	0.000716	0.000613	0.001022
		*Green*	0.000673	0.000526	−0.000036
		*Blue*	0.001876	0.000145	0.000808
	Vertical	*Red*	−0.000461	0.000217	0.002322
		*Green*	0.000380	−0.000329	−0.001155
		*Blue*	−0.001326	−0.000706	0.000292
	Diagonal	*Red*	0.000112	−0.001578	0.001852
		*Green*	0.001907	0.001407	0.001052
		*Blue*	0.000716	−0.000916	0.002042
mountain	Horizontal	*Red*	0.001664	−0.000739	0.000009
		*Green*	0.001930	0.001564	0.000449
		*Blue*	−0.004610	−0.004647	−0.000514
	Vertical	*Red*	−0.001585	−0.004933	−0.001195
		*Green*	0.001847	0.002623	−0.001546
		*Blue*	0.000481	−0.000196	0.001189
	Diagonal	*Red*	−0.004117	0.001979	0.000258
		*Green*	0.000991	−0.001514	0.001032
		*Blue*	0.003054	0.003152	−0.000282

It can be found that the performance of the proposed algorithm is nearly as good as that of the algorithm in [33]. This can be seen more clearly by comparing it with the algorithms proposed by Wang [27], Murillo-Escobar [28] and Tong [31], all in 2015 and by Wu [36] and Tong [37], both in 2016. To our knowledge, few algorithms proposed recently handle the correlation between different colors. Thus, there may be a need for a color image encryption algorithm. Because there is no recent data on correlation between different colors, the data shown in Table 10 is just the correlation between the same colors.

**Table 10:** Diagonal correlation of two adjacent pixel of Lena’s cipher with same color.

		Red	Green	Blue
proposed algorithm	Horizontal	0.0006	0.0013	−0.0011
	Vertical	−0.0001	0.0007	−0.0008
	Diagonal	−0.0004	−0.0009	−0.0001
Wang’s algorithm [27]	Horizontal	−0.0127	−0.0075	−0.0007
	Vertical	0.0067	−0.0068	0.0042
	Diagonal	0.0060	−0.0078	0.0026
Murillo-Escobar’s algorithm [28]	Horizontal	0.0135	−0.0835	−0.0170
Tong’s algorithm [31]	Horizontal	0.0015	0.0068	0.0031
	Vertical	0.0037	0.0042	0.0097
	Diagonal: Lower left to top right	0.0091	0.0130	0.0179
	Diagonal: Lower right to top left	0.0093	0.0083	0.0087
Wu’s algorithm [36]	Horizontal	−0.0206	−0.0005	0.0016
	Vertical	−0.0116	0.0002	0.0133
	Diagonal	0.0097	0.0189	−0.0123
Tong’s algorithm [37]	Horizontal	−0.0104	−0.0029	0.0124
	Vertical	0.0096	0.0127	0.0117
	Diagonal	0.0216	0.0135	0.0304

Results show that different colors in different directions have little correlation and the proposed algorithm has better correlation performance than the other recent algorithms.

### NPCR and UACI

The Number of Pixels Change Rate (NPCR) indicates the rate of the number of pixels that change when one pixel in the plain image is changed. As the NPCR approaches 99.6094%, the more sensitive the crypto-system is to changes in the plain image, and the more effective it is in resisting a plaintext attack. The UACI(Unified Average Changing Intensity) indicates the average intensity of differences between the plain image and the ciphered image. As The UACI approaches 33.4635%, the crypto-system becomes more effective at resisting differential attacks. NPCR and UACI values can be calculated as follows,

$$\begin{array}{c} NPCR=\frac{\sum_{ij}D\left(i,j\right)}{Width\times Hight}\times 100\% \end{array}$$ (12)

$$\begin{array}{c} UACI=\frac{1}{Width\times Hight}[\sum_{ij}\frac{\left|c_{i}\left(i,j\right)-c_{2}\left(i,j\right)\right|}{255}]\times 100\% \end{array}$$ (13)

where *c*_1_(*i*, *j*) and *c*_2_(*i*, *j*) are the cipher-image pixel values before and after one pixel of the plain image is changed. If *c*_1_(*i*, *j*) ≠ *c*_2_(*i*, *j*), *D*(*i*, *j*) = 1; otherwise, *D*(*i*, *j*) = 0.

**Fig 12:**
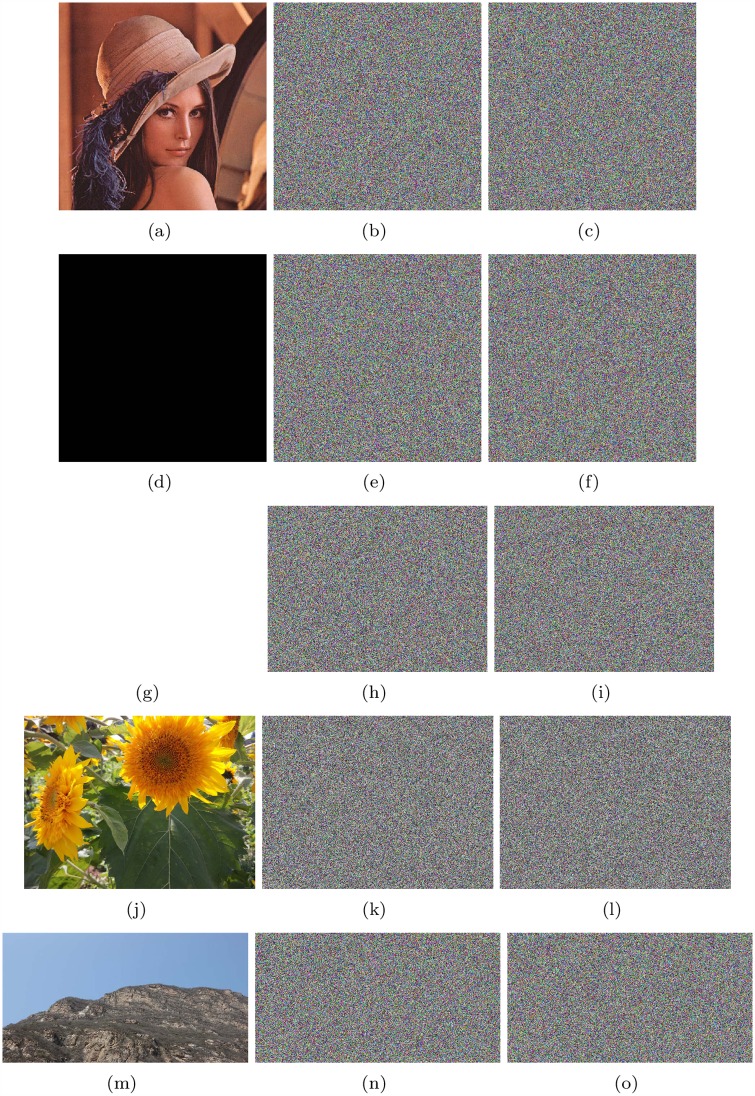
NPCR and UACI of Lena, all-zero image, white, flower and mountain. (a) Plain image Lena; (b) Encrypted image of Lena; (c) Encrypted image of Lena with a pixel changed; (d) all-zero Image; (e)Encrypted image of all-zero Image; (f)Encrypted image of all-zero with a pixel changed; (g) Plain image white; (h) Encrypted image of white; (i) Encrypted image of white with a pixel changed; (j) Plain image flower; (k) Encrypted image of flower; (l) Encrypted image of flower with a pixel changed; (m) Plain image mountain; (n) Encrypted image of mountain; (o) Encrypted image of mountain with a pixel changed.

The test figures are shown as Fig 12, and the results are shown in Table 11 and Table 12.

**Table 11:** NPCR and UACI of Lena.

		Red	Green	Blue
proposed algorithm	NPCR	99.6006%	99.6178%	99.5975%
	UACI	33.4418%	33.5298%	33.4927%
Liu’s algorithm [26]	NPCR	99.6231%	99.6338%	99.6170%
	UACI	33.4747%	33.5683%	33.3382%
Murillo-Escobar’s algorithm [28]	NPCR	99.63%	99.60%	99.61%
	UACI	33.31%	33.34%	33.43%
Wu’s algorithm [36]	NPCR	99.6101%	99.6136%	99.6141%
	UACI	33.4695%	33.4643%	33.4665%

**Table 12:** NPCR and UACI of all-zero, white, flower and mountain.

		Red	Green	Blue
all-zero	NPCR	99.6117%	99.6223%	99.6201%
	UACI	33.5307%	33.3785%	33.5307%
white	NPCR	99.6251%	99.6144%	99.6119%
	UACI	33.3961%	33.4915%	33.4136%
flower	NPCR	99.6112%	99.5928%	99.6026%
	UACI	33.4735%	33.4868%	33.4600%
mountain	NPCR	99.6001%	99.5866%	99.6074%
	UACI	33.4313%	33.4872%	33.5898%

These results show the good diffusion property of the algorithm. In addition, this indicates that the algorithm can resist plain-text attacks and differential attacks.

### Sensitivity to cipher image

**Fig 13:**
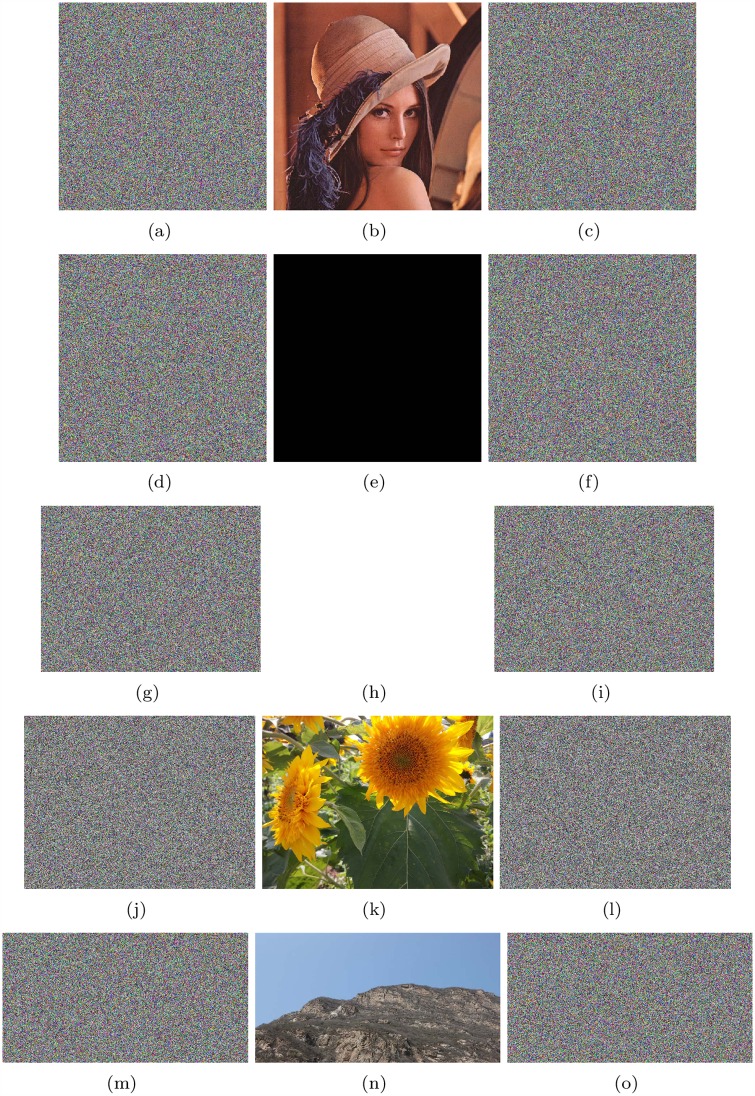
Sensitivity to cipher image test results of Lena, all-zero image, white, flower and mountain. (a) cipher of Lena; (b) decrypted image of Lena’s cipher; (c) decrypted image of Lena’s cipher with a pixel changed; (d) cipher of all-zero; (e) decrypted image of all-zero’s cipher; (f) decrypted image of all-zero’s cipher with a pixel changed; (g) cipher of white; (h) decrypted image of white’s cipher; (i) decrypted image of white’s cipher with a pixel changed; (j) cipher of flower; (k) decrypted image of flower’s cipher; (l) decrypted image of flower’s cipher with a pixel changed; (m) cipher of mountain; (n) decrypted image of mountain’s cipher; (o) decrypted image of mountain’s cipher with a pixel changed.

As done in [13], when one pixel of cipher image is changed, the recovered plain image exhibits no correlation to the plain image, then the cipher can resist a chosen-cipher attack. Similarly, the NPCR and correlation between the recovered image, with one pixel of the cipher image changed, and the plain image are computed to prove the proposed algorithm’s resistance to the chosen-cipher attack. Test figures are shown as Fig 13, and results are shown in Table 13.

**Table 13:** Correlation between Fig 13(b) and 13(c).

	Red	Green	Blue
*Red*	−0.0026	−0.0006	0.0016
*Green*	−0.0012	0.0008	0.0018
*Blue*	0.0001	0.0010	0.0017

The results show that the proposed algorithm can resist a chosen-cipher attack.

### Information entropy

Information entropy of a cipher image can be computed as Eq (14).

$$\begin{array}{c} H\left(m\right)=\sum_{i=0}^{2^{N}-1}p\left(m_{i}\right)log_{2}\frac{1}{p(m_{i})} \end{array}$$ (14)

where *p*(*m*_*i*_) represents the probability of symbol *m*_*i*_, and *log*_2_ represents the base 2 logarithm so that the entropy is expressed in bits, *N* represents the number of bits used to represent a pixel.

For one color channel of a pixel, it’s clear that *N* = 8. If an image is ideally random, for each *i*, *p*(*m*_*i*_) = 1/256. Thus, the ideal value of *H*(*m*) is 8. The information entropy of ciphers encrypted by the proposed algorithm is shown in Tables 14 and 15.

**Table 14:** Information Entropy of Lena’s cipher.

	Red	Green	Blue
proposed algorithm	7.999341	7.999337	7.999245
algorithm in [33]	7.999369	7.999299	7.999319
Wang’s algorithm [27]	7.9974	7.9970	7.9971
Murillo-Escobar’s algorithm [28]	7.9974	7.9975	7.9969
Wu’s algorithm [36]	7.9914	7.9907	7.9907

**Table 15:** Information Entropy of all-zero’s, white’s, flower’s and mountain’s cipher.

	Red	Green	Blue
all-zero	7.999258	7.999278	7.999282
white	7.999321	7.999447	7.999417
flower	7.999814	7.999762	7.999743
mountain	7.999210	7.999319	7.999222

The results show that the entropy of all the three color channels is close to the ideal value of 8. Thus, the algorithm is secure against entropy attacks.

### Key sensitivity

**Fig 14:**
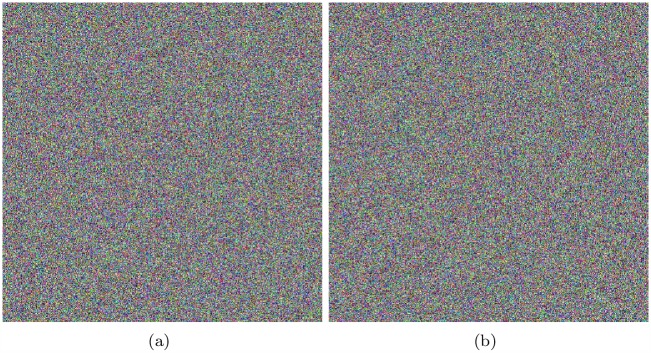
Encryption key sensitivity showed by Lena. (a) cipher of Lena; (b) cipher of Lena with key $x_{1}^{*}$ changed 10^−15^.

**Fig 15:**
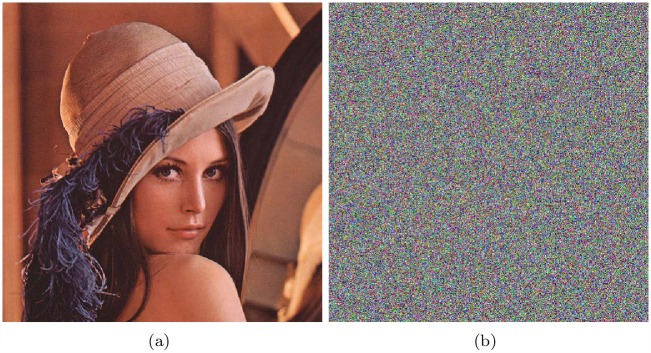
Decryption key sensitivity showed by Lena. (a) Decrypted image of Lena’s cipher; (b) Decrypted image of Lena’s cipher with key $x_{1}^{*}$ changed 10^−15^.

Key sensitivity is a simple way to find out the size of the key space [14]. In this test, we adjust keys in double form by changing the last number of the decimal and adjust integer keys by adding 1. Then, the correlation between the ciphers before and after the change to the key will be computed. This is done for both the encryption process and the decryption process (shown as Figs 14 and 15).

The original keys are $x_{1}^{*}=3.05152212424679$, $x_{2}^{*}=1.58254212245123$, $x_{3}^{*}=15.6238853231785$, $x_{4}^{*}=4.78999921123234$, $x_{5}^{*}=1.98243221252248$, $x_{6}^{*}=14.2532112455785$, *M*_0_ = 20 and *N*_0_ = 30.

The results for encryption key sensitivity are shown in Table 16.

**Table 16:** The results for encryption key sensitivity.

	Red	Green	Blue	Red	Green	Blue
	$x_{1}^{*}=3.05152212424670$, the others rest invariant.			$x_{2}^{*}=1.58254212245124$, the others rest invariant.		
*Red*	−0.0003	0.0011	− 0.0001	0.0004	−0.0012	0.0024
*Green*	−0.0023	−0.0042	−0.0007	0.0020	−0.0007	−0.0022
*Blue*	0.0012	−0.0003	−0.0007	−0.0018	−0.0001	0.0040
	$x_{3}^{*}=15.6238853231786$, the others rest invariant.			$x_{4}^{*}=4.78999921123235$, the others rest invariant.		
*Red*	−0.0006	0.0031	−0.0014	−0.0023	−0.0016	−0.0001
*Green*	0.0027	0.0002	0.0024	0.0008	0.0021	0.0014
*Blue*	0.0021	−0.0002	0.0036	−0.0008	−0.0005	−0.0030
	$x_{5}^{*}=1.98243221252249$, the others rest invariant.			$x_{6}^{*}=14.2532112455786$, the others rest invariant.		
*Red*	0.0001	0.0008	0.0013	0.0035	−0.0024	0.0001
*Green*	0.0028	−0.0016	0.0021	0.0028	−0.0008	0.0029
*Blue*	0.0013	−0.0020	−0.0054	0.0025	0.0004	−0.0020
	*M*_0_ = 21, the others rest invariant.			*N*_0_ = 31, the others rest invariant.		
*Red*	0.0015	−0.0029	0.0013	0.0022	−0.0016	0.0012
*Green*	0.0007	−0.0037	0.0017	−0.0028	−0.0012	−0.0005
*Blue*	0.0035	−0.0023	−0.0010	−0.0015	0.0001	0.0014

The results for decryption key sensitivity show as Table 17.

**Table 17:** The results for decryption key sensitivity.

	Red	Green	Blue	Red	Green	Blue
	$x_{1}^{*}=3.05152212424670$, the others rest invariant.			$x_{2}^{*}=1.58254212245124$, the others rest invariant.		
Red	−0.0002	0.0010	0.0016	−0.0020	−0.0023	−0.0001
Green	−0.0013	0.0004	0.0021	−0.0012	−0.0016	0.0003
Blue	−0.0004	−0.0004	0.0014	0.0001	−0.0002	0.0004
	$x_{3}^{*}=15.6238853231786$, the others rest invariant.			$x_{4}^{*}=4.78999921123235$, the others rest invariant.		
Red	−0.0026	0.0011	−0.0015	0.0021	−0.0018	0.0011
Green	−0.0035	0.0013	−0.0025	0.0020	−0.0018	0.0012
Blue	−0.0025	0.0011	−0.0027	0.0013	−0.0026	0.0005
	$x_{5}^{*}=1.98243221252249$, the others rest invariant.			$x_{6}^{*}=14.2532112455786$, the others rest invariant.		
Red	−0.0012	−0.0005	0.0013	0.0009	0.0001	−0.0053
Green	−0.0020	−0.0014	0.0026	0.0015	0.0004	−0.0042
Blue	−0.0020	−0.0023	0.0035	0.0021	−0.0003	−0.0027
	*M*_0_ = 21, the others rest invariant.			*N*_0_ = 31, the others rest invariant.		
Red	0.0009	0.0020	0.0016	−0.0017	−0.0030	0.0003
Green	0.0017	0.0024	0.0016	−0.0023	−0.0034	0.0003
Blue	0.0020	0.0031	0.0021	−0.0023	−0.0028	0.0002

Thus, we have shown that the modified chaotic algorithm is sensitive to small key changes, such that a small change in the key will generate a completely different decryption result and cannot be used to find the correct plain image.

### Key space analysis

In the previous subsection, it us shown that a key in double form has a precision of 10^−15^. As mentioned in the beginning of this section, there are 6 doubles and 2 integers used as keys for the proposed algorithm. Even if integers are ignored, the key space, composed of all 6 double numbers, is greater than 10^15*6^ > 2^300^. Thus, the key space is large enough to resist a brute-force attack.

## Speed analysis

Compared with the algorithm proposed in [33], which also uses the 4-pixel Feistel structure, this algorithm has two methods that can be used to speed it up, namely the simple round function and the modified 4-pixel Feistel structure.

**Fig 16:**
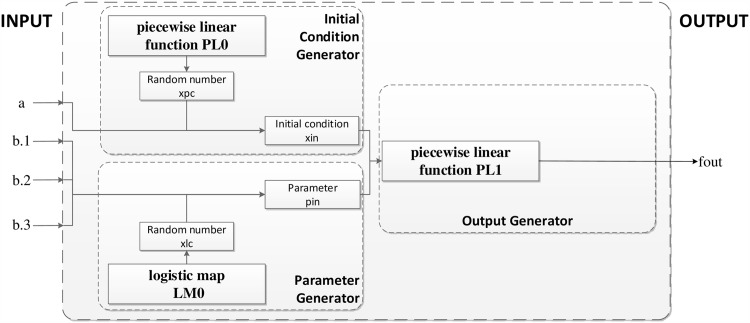
Round function of [33] for one color of one pixel.

The round function in the algorithm is simpler than the one introduced in [33], where three chaotic functions constructed one round function for one color of one pixel (Fig 16). For each round of [33], 12 piecewise linear functions and 6 logistic functions are needed. However, in our research, 6 piecewise linear functions and 8 tent maps replace the 12 piecewise linear functions and 6 logistic maps used in [33]. As a result, the computation for each round function becomes easier.

**Fig 17:**
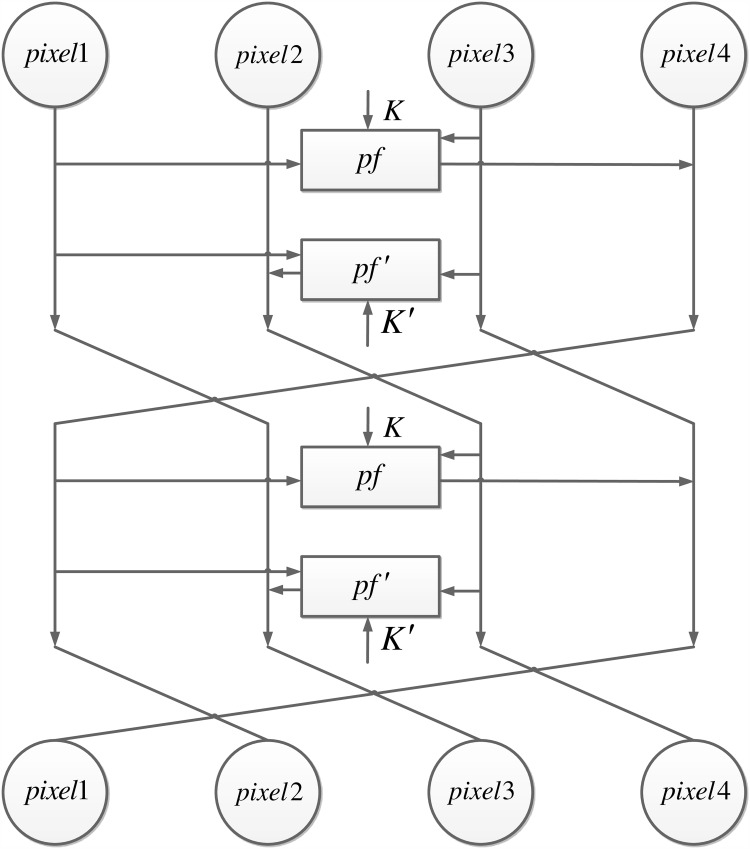
Two round 4-pixel Feistel structure of [33].

The second method used to speed up the algorithm is the modified 4-pixel Feistel structure. It can easily be found that the algorithm given in [33] can be divided into 2 independent parts each of which has a 2-round Feistel structure (Fig 17) in the 2 times of dependent encryption progress. The use of these two methods means that slightly different functions are used. If these two functions are not secure, the dependent encryption progress will be meaningless. As a result, we attempt to classify the security properties of these two functions.

**Fig 18:**
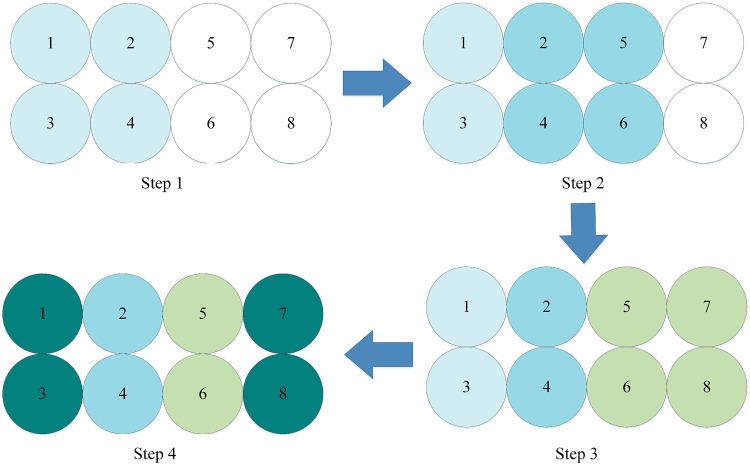
First iteration of dependent encryption using original 4-pixel Feistel structure.

The first time of dependent encryption progress can be presented in Fig 18 (8-pixel example). Due to the good properties of round functions based on the multiple chaotic system, the two encrypted pixels can be treated as result of two other pixels, but independent from each other. For an image of 8 pixels, using the algorithm in [33] requires 4 steps to complete the first iteration of the dependent encryption process. A Feistel cipher with two rounds can handle the four pixels so that the position (or diffusion) will not be shown in the image. In this image, the deeper color of the pixel indicates that more pixels affect the handling of this pixel.

It is found that for the four pixels handled in step 1, each is affected by all four pixels. However, for the first 4 pixels handled in step 2, they are affected by the first six pixels. In step 3, the 4 pixels are affected by all 8 pixels in the image. Step 4 extends the affect to pixel 1 and pixel 3. After the first iteration of the dependent encryption, only a few pixels at the beginning and end of the image should be affected by all the pixels. A change of one pixel should affect every pixel in the image, which means that the NPCI and UACR values will be good, which is necessary for a secure image encryption algorithm. Thus, a second iteration of the dependent encryption algorithm is necessary to extend the affect to all pixels in the image. In brief, in the first iteration of dependent encryption the effect of pixels accumulates. During the second iteration of dependent encryption the affect extends through the whole image.

As shown in the analysis above, the first iteration of dependent encryption aims at accumulating the effect of the encryption on the pixels. Thus, a simpler structure(Fig 2)is proposed, so that the accumulation of pixels is just realized by changing the twist direction without encrypting all pixels.

**Fig 19:**
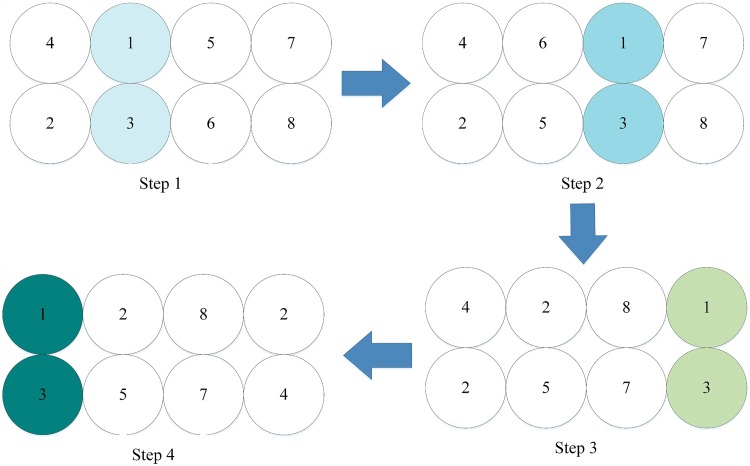
First iteration of dependent encryption using modified 4-pixel Feistel structure (only one round considered).

The structure of this method uses the same functions as the original structure, but the computations are more simple. In the first iteration of the dependent encryption, if only the first round with round functions is considered, only two pixels are handled. The affect is propagated as progress continues, as shown in Fig 19. The second round occurs without using round functions and provides a way to extend the results from two pixels to all four pixels in a handled block.

During the second iteration of dependent encryption, only an extension of this affect is needed. This is accomplished using the 1-round Feistel, which is described below. If round functions aren’t taken into count (in effect, simpler functions have been proposed), the operations needed for the modified 4-pixel Feistel structure are only half of the original amount, in both the encryption phase and the key generation phase.

Due to the difference of computer configurations, code optimization, or even image format, the speed of an algorithm is difficult to compare exactly. Some results are shown in Table 18 for reference. As shown, the proposed algorithm is twice as fast as that in [33], which also uses 4-pixel Feistel structure. We also show that our algorithm performs better than those developed in other recent research.

**Table 18:** Speed analysis result (in Seconds).

	Software platform	Hardware platform	Image size	
			256*256	512*512
proposed algorithm	VS 2012	Intel Q9500 2.83GHz	0.15	0.58
algorithm in [33]	VS 2012	Intel i5-4210U 1.70GHz	0.37	1.38
Ping’s algorithm [38]	Mathematica 8.0	Intel Pentium Dual Core 2.9GHz	1.18	–
Faraoun’s algorithm [39]	Delphi 6	Intel i7-2600 3.40GHz	–	0.75

Through our analysis and simulation, our two methods (the simple round function and modified 4-pixel Feistel structure) are shown to accelerate the proposed algorithm to much faster speeds than those found in [33], which also uses 4-pixel Feistel structure, as well as those found in other recent research.

## Conclusions

In our research we propose a fast color image encryption algorithm using 4-pixel Feistel structure and multiple chaotic systems. We perform multiple simulation experiments, including histogram and key space and speed analysis, measure the correlation of two adjacent pixels, and find the NPCR, UACI, information entropy, and key sensitivity values to show that the proposed algorithm has good statistical and diffusion properties and can resist many different types of attacks. In our analysis we give two methods that are used to speed up the algorithm. First, we introduce the use of a simple round function, based on a piecewise linear function, and a tent map is proposed to reduce computational cost during each round. Second, a modified 4-pixel Feistel structure, which reduces round numbers by changing twist direction in a secure way, is proposed to help the algorithm proceed in an efficient way. Simulation results prove that these two methods increase the speed of the proposed algorithm to twice that of a similarly structured algorithm (A 256*256 image can be encrypted in 0.15s compared with 0.37s) and even more for other recently developed algorithms.
